# Association between preoperative neutrophil–lymphocyte ratio and mortality after plastic and reconstructive surgery

**DOI:** 10.1038/s41598-021-00901-7

**Published:** 2021-11-02

**Authors:** Ha Min Sung, Seung-Hwa Lee, Ah Ran Oh, Sojin Kim, Jeayoun Kim, Joonhee Gook, Jae Ni Jang, Jungchan Park

**Affiliations:** 1 Link Plastic Surgery Clinic, Seoul, South Korea; 2 Division of Cardiology, Department of Medicine, Samsung Medical Center, Sungkyunkwan University School of Medicine, Heart Vascular Stroke Institute, Seoul, South Korea; 3 Department of Biomedical Engineering, Seoul National University College of Medicine, Seoul, South Korea; 4 Department of Anesthesiology and Pain Medicine, Samsung Medical Center, Sungkyunkwan University School of Medicine, 81 Irwon-ro, Gangnam-gu, Seoul, 06351 South Korea; 5 Department of Anesthesiology and Pain Medicine, Kangwon National University Hospital, Chuncheon, South Korea; 6 Department of Biomedical Sciences, Ajou University Graduate School of Medicine, Suwon, South Korea

**Keywords:** Biomarkers, Medical research

## Abstract

Predictive factors associated with postoperative mortality have not been extensively studied in plastic and reconstructive surgery. Neutrophil–lymphocyte ratio (NLR), a systemic inflammation index, has been shown to have a predictive value in surgery. We aimed to evaluate association between preoperative NLR and postoperative outcomes in patients undergoing plastic and reconstructive surgery. From January 2011 to July 2019, we identified 7089 consecutive adult patients undergoing plastic and reconstructive surgery. The patients were divided according to median value of preoperative NLR of 1.84. The low NLR group was composed of 3535 patients (49.9%), and 3554 patients (50.1%) were in the high NLR group. The primary outcome was mortality during the first year, and overall mortality and acute kidney injury were also compared. In further analysis, outcomes were compared according to quartile of NLR, and a receiver operating characteristic curve was constructed to estimate the threshold associated with 1-year mortality. This observational study showed that mortality during the first year after plastic and reconstructive surgery was significantly increased in the high NLR group (0.7% vs. 3.5%; hazard ratio, 4.23; 95% confidence interval, 2.69–6.63; *p* < 0.001), and a graded association was observed between preoperative NLR and 1-year mortality. The estimated threshold of preoperative NLR was 2.5, with an area under curve of 0.788. Preoperative NLR may be associated with 1-year mortality after plastic and reconstructive surgery. Further studies are needed to confirm our findings.

## Introduction

The applications of plastic and reconstructive surgery are various, and surgical procedures are complicated. On the other hand, these surgeries are generally considered relatively safe procedures with low risk of mortality. Compared to other major surgeries, the risk factors related to perioperative mortality in plastic and reconstructive surgery have not been extensively investigated^1^.

Neutrophil–lymphocyte ratio (NLR) is a simple index that reflects the systemic inflammatory response^2^. It has shown predictive value in various acute and chronic situations, including cardiovascular disease, liver cirrhosis, and cancer^2–8^. A high preoperative NLR has been reported to be associated with postoperative outcomes^9^. Specifically, these associations with NLR were demonstrated for mortality and acute kidney injury which could be found even after minor procedures^10–12^. However, these findings were demonstrated in limited types of surgeries, such as mortality after cardiac surgery or for recurrence after cancer resection^9,13–17^. Furthermore, neither the generalizability of the association between perioperative NLR and postoperative outcome nor the normal range of NLR for surgical patients has been fully established. Therefore, in this study, we aimed to evaluate the association between NLR and postoperative outcome in patients undergoing plastic and reconstructive surgery, a surgical subspecialty in which NLR has gained relatively less attention. In addition, we estimated the threshold of preoperative NLR level that was associated with mortality. Our findings may be helpful for clinicians to predict postoperative outcome using a simple index that can be calculated using only the results of a differential blood cell count.

## Results

### Baseline characteristics

From January 2011 to July 2019, a total of 7089 patients underwent plastic and reconstructive surgery and had an available preoperative NLR value. We divided the study patients into two groups according to median NLR (1.84): 3535 patients (49.9%) in the low NLR group and 3554 patients (50.1%) in the high NLR group. The patients’ baseline characteristics are summarized in Table 1. The high NLR group tended to be male and had higher creatinine level and Charlson comorbidity index score but shorter operative duration.

**Table 1:** Baseline characteristics according to the median value of preoperative NLR.

	Entire population				Propensity score matched population			
	Low NLR (N = 3535)	High NLR (N = 3554)	*p* value	ASD	Low NLR (N = 3305)	High NLR (N = 3305)	*p* value	ASD
Preoperative NLR	1.33 (± 0.49)	3.49 (± 3.72)			1.32 (± 0.32)	3.37 (± 2.99)		
Age (years)^a^	46 (37–55)	45 (36–55)	0.8	0.6	46 (36–54)	44 (35–54)	0.01	2.3
Male^a^	1002 (28.3)	1164 (32.8)	< 0.001	9.6	959 (29.0)	1019 (30.8)	0.11	4
Smoking^a^	352 (10.0)	386 (10.9)	0.21	3	337 (10.2)	343 (10.4)	0.84	0.6
Alcohol^a^	925 (26.2)	849 (23.9)	0.03	5.3	812 (24.6)	790 (23.9)	0.55	1.6
Hypertension^a^	165 (4.7)	246 (6.9)	< 0.001	9.7	160 (4.8)	173 (5.2)	0.5	1.8
**Charlson comorbidity index**^a^	0.31 (± 0.96)	0.42 (± 1.16)	< 0.001	9.7	0.32 (± 0.96)	0.34 (± 1.02)	0.42	2
Myocardial infarction	6 (0.2)	6 (0.2)			5 (0.2)	3 (0.1)		
Heart failure	8 (0.2)	17 (0.5)			7 (0.2)	9 (0.3)		
Peripheral vascular disease	9 (0.3)	10 (0.3)			9 (0.3)	6 (0.2)		
Cerebrovascular disease	49 (1.4)	61 (1.7)			48 (1.5)	50 (1.5)		
Dementia	0	1 (0.0)			1 (0.0)	1 (0.0)		
Chronic pulmonary disease	1 (0.0)	3 (0.1)			1 (0.0)	1 (0.0)		
Rheumatic disease	13 (0.4)	50 (1.4)			13 (0.4)	37 (1.1)		
Peptic ulcer disease	0	0			0	0		
Mild liver disease	117 (3.3)	127 (3.6)			107 (3.2)	100 (3.0)		
Diabetes without complication	94 (2.7)	174 (4.9)			89 (2.7)	122 (3.7)		
Diabetes with complication	36 (1.0)	70 (2.0)			33 (1.0)	42 (1.3)		
Hemiplegia	8 (0.2)	13 (0.4)			8 (0.2)	7 (0.2)		
Renal disease	28 (0.8)	86 (2.4)			25 (0.8)	55 (1.7)		
Any malignancy	192 (5.4)	162 (4.6)			185 (5.6)	152 (4.6)		
Moderate to severe liver disease	0	5 (0.1)			0	1 (0.0)		
Metastatic solid tumor	0	0			0	0		
Human immunodeficiency virus	0	1 (0.0)			0	0		
**Preoperative blood test**								
Hemoglobin (g/dL)^a^	13.11 (± 1.84)	13.14 (± 1.81)	0.5	1.3	13.14 (± 1.80)	13.14 (± 1.78)	0.92	0.2
Creatinine (mg/dL)^a^	0.78 (± 0.42)	0.83 (± 0.68)	< 0.001	9.1	0.78 (± 0.39)	0.79 (± 0.46)	0.44	1.9
**Operative variables**								
Duratrion (h)^a^	3.33 (1.58–4.85)	3.18 (1.33–4.66)	< 0.001	8.8	3.28 (1.55–4.78)	3.37 (1.62–4.78)	0.63	2.1
General anesthesia^a^	3385 (95.8)	3212 (90.4)	< 0.001	21.3	3158 (95.6)	3157 (95.5)	> 0.99	0.1
**Type**								
Head and neck	514 (14.5)	512 (14.4)			476 (14.4)	471 (14.3)		
Trunk and extremities	649 (18.4)	693 (19.3)			600 (18.2)	641 (19.4)		
Breast	1607 (45.5)	1610 (45.3)			1510 (45.7)	1507 (45.6)		
Aesthetic surgery	97 (2.7)	73 (2.1)			88 (2.7)	67 (2.0)		
Mass excision	668 (18.9)	666 (18.7)			631 (19.1)	619 (18.7)		

Values are n (%), mean (± standardized difference), or median (interquartile range).

*ASD* absolute standardized mean difference, *NLR* neutrophil-to-lymphocyte ratio.

^a^Following variables were retained for propensity score matching.

**Figure 1:**
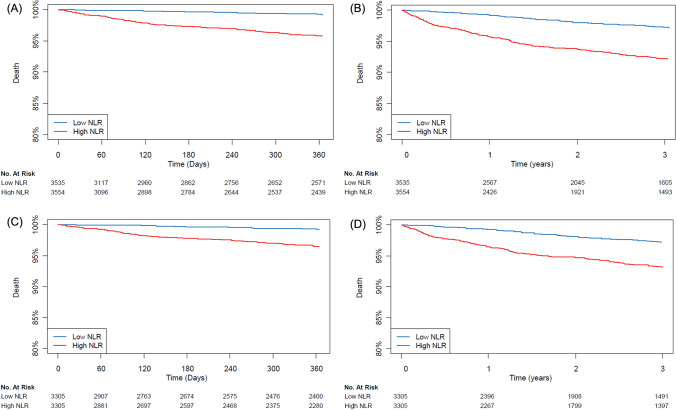
Kaplan Meier curves of the groups divided by median value for mortality in the entire population during (**A**) 1-year and (**B**) overall follow-up periods, and propensity matched population during (**C**) 1-year and (**D**) overall follow-up periods.

**Figure 2:**
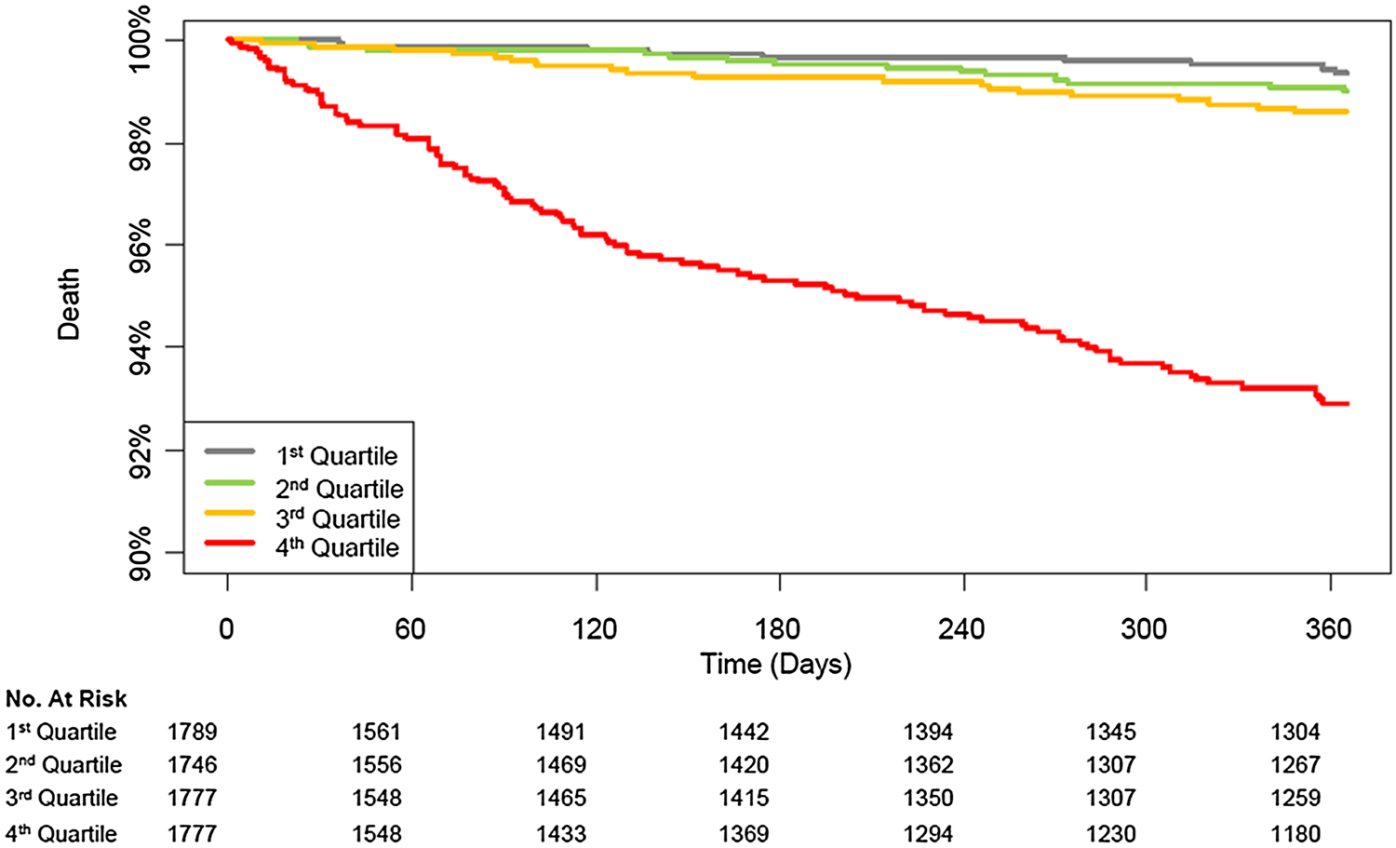
Kaplan Meier curves of the groups divided by quartile for mortality during 1-year.

**Figure 3:**
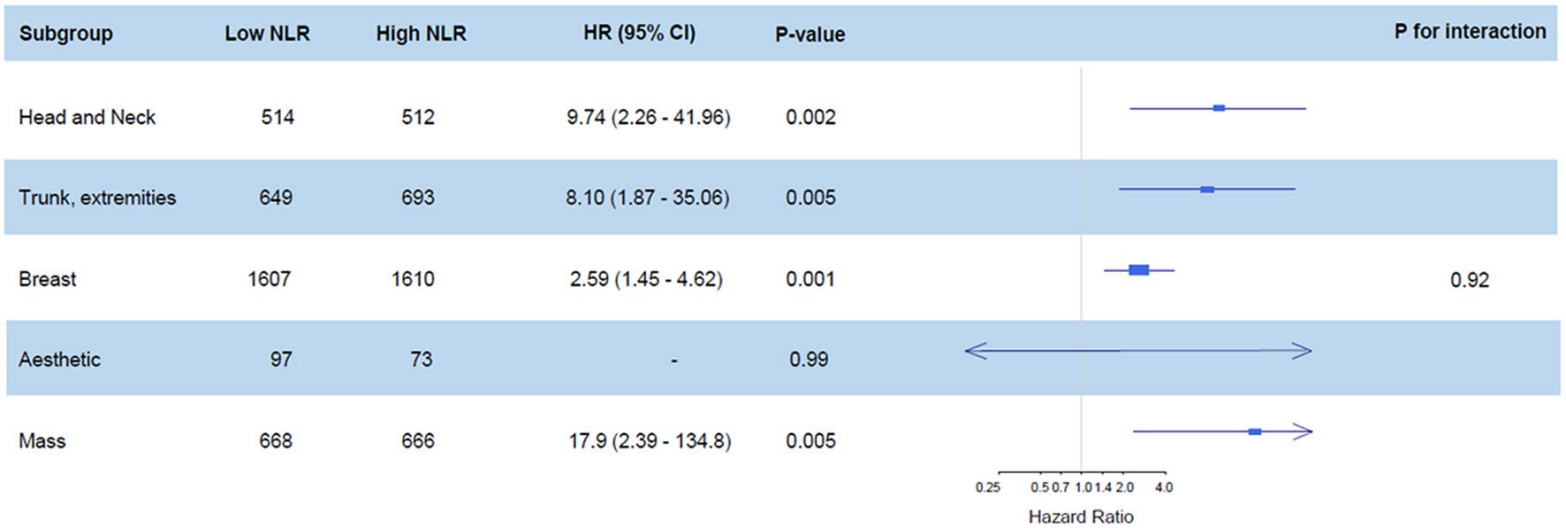
Subgroup analysis according to types of plastic and reconstructive surgery.

**Figure 4:**
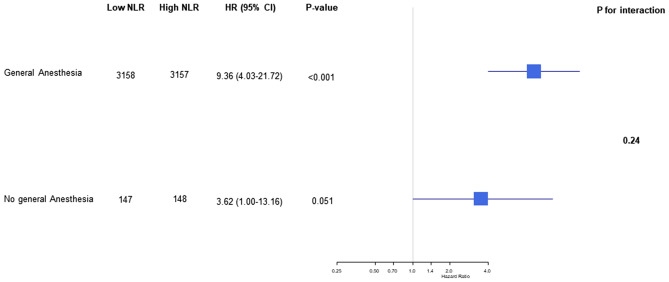
Subgroup analysis according to general anesthesia.

The mortality rate was 2.1% (149/7089) during the first year after surgery and 4.9% (348/7089) during the overall follow-up period. The median follow-up duration was 365 days (interquartile range: 258–365 days) for 1-year mortality and 902 days (interquartile range: 258–1717 days) during the overall follow-up period. After an adjustment, mortality during the first year was significantly higher in the high NLR group (0.7% vs. 3.5%; HR, 4.23; 95% CI 2.69–6.63; *p* < 0.001) (Table 2). Similar results were found for mortality during the overall follow-up period (3.0% vs. 6.8%; HR, 2.06.; 95% CI 1.64–2.60; *p* < 0.001) and for postoperative acute kidney injury (0.3% vs. 1.5%; OR, 3.38; 95% CI 1.74–6.59; *p* < 0.001) After dividing patients into four groups according to quartile of NLR, the 1-year mortalities of patients in the 3rd and 4th quartile groups were higher than those of patients in the 1st quartile group (0.5% vs. 1.1%; HR, 1.51.; 95% CI 0.02–2.23; *P* = 0.04 for the 3rd quartile group and 0.5% vs. 6.0%; OR, 2.30; 95% CI 1.84–2.89; *p* < 0.001 for the 4th quartile group) (Table 3). Survival curves are presented in Figs. 1 and 2. The subgroup analysis was conducted according to types of surgery and general anesthesia (Figs. 3 and 4), and there was no significant interaction.

**Table 2:** Clinical outcomes according to the median value of preoperative NLR.

	Low	High	Unadjusted HR/OR (95% CI)	*p* value	Adjusted HR/OR (95% CI)	*p* value
**Entire population**	N = 3535	N = 3554				
One-year mortality	23 (0.7)	126 (3.5)	5.59 (3.59–8.71)	< 0.001	4.23 (2.69–6.63)	< 0.001
Overall mortality	106 (3.0)	242 (6.8)	2.39 (1.90–3.00)	< 0.001	2.06 (1.64–2.60)	< 0.001
Acute kidney injury						
Any	12 (0.3)	55 (1.5)	4.62 (2.47–8.63)	< 0.001	3.38 (1.74–6.59)	< 0.001
Stage 1	11 (0.3)	48 (1.4)	4.39 (2.27–8.46)	< 0.001	3.05 (1.51–6.17)	0.002
Stage 2	1 (0.0)	7 (0.2)	6.97 (0.86–56.72)	0.07	6.87 (0.83–56.90)	0.07
**Propensity score matched population**	N = 3305	N = 3305				
One-year mortality	21 (0.6)	96 (2.9)			13.17 (6.98–24.82)	< 0.001
Overall mortality	96 (2.9)	193 (5.8)			10.82 (6.38–18.35)	< 0.001
Acute kidney injury						
Any	10 (0.3)	35 (1.1)			3.21 (1.68–6.63)	< 0.001
Stage 1	9 (0.3)	30 (0.9)			3.02 (1.53–6.51)	0.003
Stage 2	1 (0.0)	5 (0.2			5.01 (0.81–95.9)	0.14

Mortalities were reported with HR, and acute kidney injury was reported with OR.

Multivariable analysis retained age, male, alcohol, preoperative creatinine level, Charlson comorbidity index, operative duration, and general anesthesia.

Propensity score matching analysis retained age, male, smoking, alcohol, preoperative creatinine/hemoglobin levels, hypertension, Charlson comorbidity index, operative duration, and general anesthesia.

*NLR* neutrophil-to-lymphocyte ratio, *HR* hazard ratio, *OR* odds ratio.

**Table 3:** Clinical outcomes according to quartile value of preoperative NLR.

	1st quartile (N = 1787)	2nd quartile (N = 1737)	3rd quartile (N = 1769)	4th quartile (N = 1737)
NLR range	< 1.35	< 1.84	< 2.61	
One-year mortality, number (%)	9 (0.5)	14 (0.8)	20 (1.1)	106 (6.0)
Unadjusted HR (95% CI)	Ref	1.59 (0.69–3.66)	1.51 (0.02–2.23)	2.30 (1.84–2.89)
*p* value		0.28	0.04	< 0.001
Overall mortality, number (%)	51 (2.9)	55 (3.2)	67 (3.8)	175 (9.8)
Unadjusted HR (95% CI)	Ref	1.12 (0.76–1.64)	1.16 (0.96–1.39)	1.56 (1.40–1.73)
*p* value		0.56	0.12	< 0.001
**Acute kidney injury**				
Any, number (%)	3 (0.2)	9 (0.5)	10 (0.6)	45 (2.5)
Unadjusted OR (95% CI)	Ref	3.09 (0.83–11.41)	1.84 (0.96–3.50)	2.49 (1.69–3.68)
*p* value		0.09	0.07	< 0.001
Stage 1, number (%)	2 (0.1)	9 (0.5)	8 (0.5)	40 (2.3)
Unadjusted OR (95% CI)	Ref	4.63 (1.00–21.46)	2.01 (0.93–4.37)	2.74 (1.71–4.40)
*p* value		0.05	0.08	< 0.001
Stage 2, number (%)	1 (0.1)	0	2 (0.1)	5 (0.3)
Unadjusted OR (95% CI)	Ref		1.42 (0.43–4.72)	1.72 (0.84–3.51)
*p* value			0.57	0.14

*NLR* neutrophil-to-lymphocyte ratio, *HR* hazard ratio, *OR* odds ratio.

**Figure 5:**
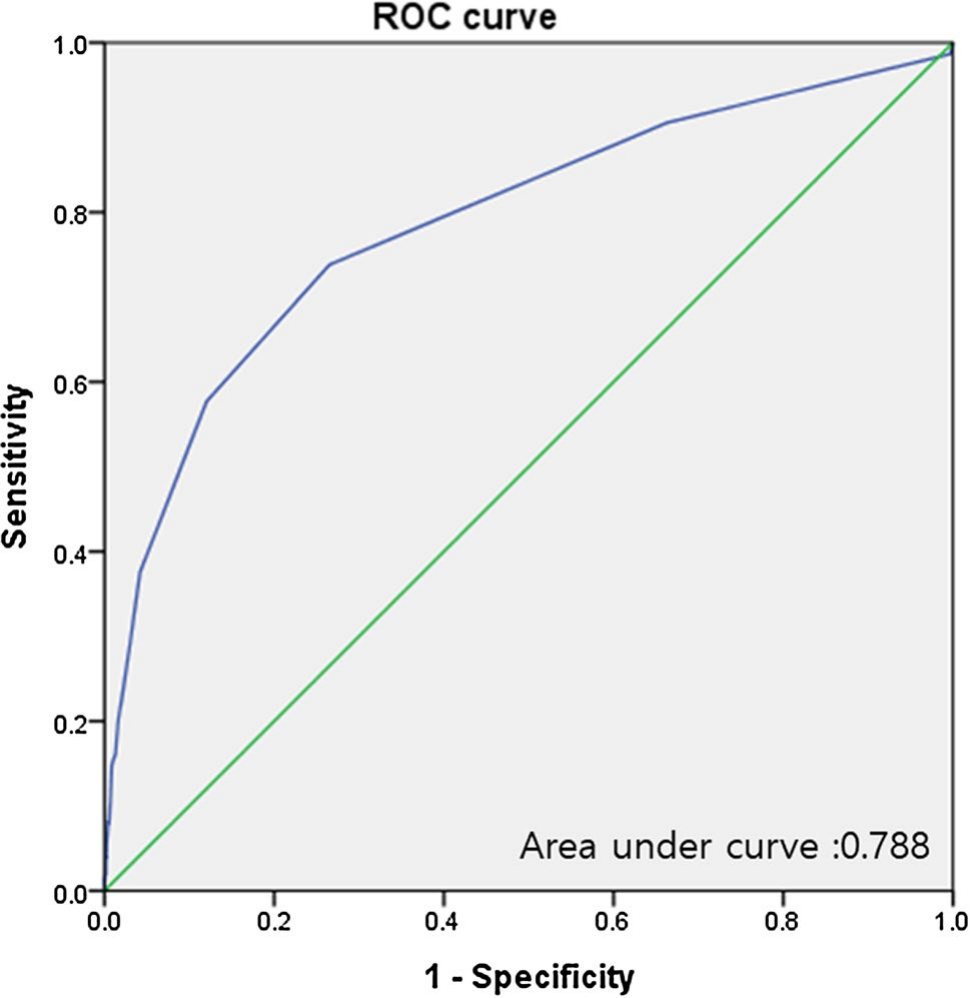
Receiver-operating characteristic plots of neutrophil–lymphocyte ratio associated with 1-year mortality for plastic and reconstructive surgery.

The threshold for preoperative NLR associated with 1-year mortality for plastic and reconstructive surgery was estimated to be 2.5 in ROC analysis. The area under the ROC curve was 0.788; based on this value, sensitivity and specificity were 73.8% and 73.5%, respectively (Fig. 5).

## Discussion

The main finding of this study was that preoperative NLR was associated with mortality and acute kidney injury after plastic and reconstructive surgery. This association showed a graded response, and the threshold of preoperative NLR level associated with 1-year mortality was estimated to be 2.5. These findings indicate that preoperative NLR may be used to predict postoperative risk in plastic and reconstructive surgery.

Although plastic and reconstructive surgeries cover a broad range of sites with various procedures, little attention has been paid to patients’ preoperative status for risk prediction because patients undergoing these surgeries are relatively healthy compared to patients undergoing other major surgeries. Previously reported perioperative factors associated with one-month mortality in plastic and reconstructive surgery include age older than 65 years, inpatient surgery, hepatorenal disease, recent chemotherapy, and partial and dependent functional status^1^.

Our study showed that preoperative NLR may be associated with postoperative mortality for plastic and reconstructive surgery. By reflecting systemic inflammation, preoperative NLR has consistently shown an association with postoperative mortality in many other areas of surgery^9,13–17^. In addition, this index in clinical practice is simple, inexpensive, reproducible, and readily available by routine blood cell count measurements. Several other biomarkers identified to measure systemic inflammation consume additional time and cost^2^, and NLR may be an adequate biomarker to predict risk in plastic and reconstructive surgery, where postoperative mortality is low. As well as mortality, NLR was associated with acute kidney injury which is a common postoperative complication^12^. The incidence of acute kidney injury was reported to be more common after the major surgical procedures, reflecting the relation with systemic inflammation from surgical stress^12^. So, our finding that NLR, an indicator of systemic inflammation, is associated with both mortality and acute kidney injury can be well explained.

The normal range of NLR has not been fully established^2^. In this study, we divided the patients at the median value for main analysis and according to quartile in further analyses. In the analysis using quartile values, the HR for mortality increased in a graded manner with NLR value, but the HR of the 4th quartile group was much higher than that of the other groups. So, the threshold of NLR associated with 1-year mortality was estimated. The estimated threshold was 2.5, which is lower than most of the values reported from other studies of NLR in surgical patients^9,13–17^. This may be related to the relatively fewer underlying diseases and overall good health of the patients undergoing plastic and reconstructive surgery compared to those with other surgery. Our result suggests that mild elevation of preoperative NLR may have predictive value in plastic and reconstructive surgery. However, need for a different threshold for NLR to be applied in plastic and reconstructive surgery and the clinical efficacy of correcting high preoperative NLR before surgery could not be fully answered in this study.

Our study should be appraised considering the following limitations. First, as a single-center retrospective study, our results may have been affected by confounding factors. Despite statistical adjustments, the effects of unmeasured variables could not be adjusted. Also, due to the long study period, advancements in both surgical techniques and postoperative management could have biased the results. Second, an adequate management for patients with high NLR remains unclear. A well-designed randomized trial may be needed. Despite these limitations, this is the first study to report an association between preoperative NLR and outcomes in plastic and reconstructive surgery.

In conclusion, preoperative high NLR may be associated with increased risk of acute kidney injury and mortality after plastic and reconstructive surgery. Further studies are needed for clinical application of our findings.

## Materials and methods

This retrospective, observational, cohort study was approved by the institutional review board at Samsung Medical Center (SMC 2020-09-001), and the need for written informed consent was waived also by the institutional review board at Samsung Medical Center considering the minimal risk for participants and retrospective nature of the study. The research was conducted following the Declaration of Helsinki and reported according to the Strengthening the Reporting of Observational Studies in Epidemiology guideline.

### Study population and data collection

From January 2011 to July 2019, consecutive adult patients undergoing plastic and reconstructive surgery at Samsung Medical Center with an available preoperative NLR level were selected for this study. The study population was divided into two groups according to median preoperative NLR of 1.84. For further analysis, the study patients were divided according to quartile of NLR as < 1.35, < 1.84, < 2.61, and ≧ 2.61. Medical records of the study patients were curated in a deidentified form using the “Clinical Data Warehouse Darwin-C,” an electronic system in which investigators search and retrieve data from the electronic archive system containing more than 2.2 million surgeries, one billion laboratory findings, 100 million disease codes, and 200 million prescriptions. In this system, mortality data from other institutions are consistently validated and updated according to the National Population Registry of the Korea National Statistical Office. Medical records were manually reviewed by an independent investigator.

### Study outcomes and definitions

The primary endpoint of this study was graft failure during a 1-year follow-up period. Secondary outcomes were mortality during overall follow-up and postoperative acute kidney injury, which was defined by the Kidney Disease: Improving Global Outcomes criteria using creatinine level^18^. The Charlson comorbidity index was calculated using the 10th revision of the International Statistical Classification of Diseases and Related Health Problems (ICD-10)^19^.

### Statistical analysis

Continuous data were presented as mean ± standard deviation and compared using the t-test or the Mann–Whitney test when applicable. Differences of categorical data were compared using χ^2^ or Fishers exact test and presented as number and incidence. We used Cox regression analysis to evaluate the association between NLR and mortality and reported the hazard ratio (HR) with a 95% confidence interval (CI). Postoperative acute kidney injury was compared using logistic regression analysis and was reported as odds ratio (OR). The following clinically relevant variables, those with *p* < 0.05, were retained in the multivariable models: age, male, alcohol consumption, preoperative creatinine level, Charlson comorbidity index, operative duration, and general anesthesia. Kaplan–Meier estimates were used to construct survival curves and were compared using the log-rank test. Subgroup analyses were performed according to types of surgery and general anesthesia and presented in forest plots. We estimated the threshold of preoperative NLR to predict 1-year mortality by constructing receiver-operating characteristic (ROC) plots, and we assessed the efficacy with Pearson’s correlation coefficient. All statistical analyses were performed with SPSS 20.0 (IBM Corp., Chicago, IL). All tests were two-tailed, and *p* < 0.05 was considered statistically significant.

## Abbreviation

NLR: Neutrophil–lymphocyte ratio
